# Genetic diversity and population structure of three traditional horse breeds of Bhutan based on 29 DNA microsatellite markers

**DOI:** 10.1371/journal.pone.0199376

**Published:** 2018-06-27

**Authors:** Jigme Dorji, Sonam Tamang, Tshewang Tshewang, Tshering Dorji, Tashi Yangzome Dorji

**Affiliations:** National Biodiversity Centre, Serbithang, Ministry of Agriculture and Forests, Thimphu, Bhutan; University of Illinois, UNITED STATES

## Abstract

The genetic variability and population structure of three Bhutanese traditional horse breeds were assessed through genotyping of 74 horses (Boeta 25, Sharta 14 and Yuta 35) for 29 microsatellite DNA loci. Altogether, 282 alleles were detected across 29 polymorphic loci. The allelic diversity (*N*_E_) (Boeta 4.94; Sharta 4.65; Yuta 5.30) and gene diversities (*H*_E_) (Boeta 0.78; Sharta 0.77; Yuta 0.79) were high. None of the breeds deviated significantly from the Hardy-Weinberg equilibrium. There was no sign of significant population bottleneck for all the breeds. The inbreeding estimates (*F*_IS_) of the breeds were low (Boeta 0.023; Sharta 0.001; Yuta 0.021). Analysis of molecular variance showed 0.6% of the total genetic variation among breeds, 1.9% among individuals and 97.5% within individuals. The global *F*_IT_, *F*_ST,_ and *F*_IS_ estimates for the population were 0.025, 0.006 and 0.019 respectively. The analysis of population structure failed to distinguish subpopulations in traditional horses and this was supported by a high genetic exchange among the breeds. Overall, the results of this study suggest a rich genetic diversity in the traditional horse despite a very low genetic differentiation among the breeds in Bhutan.

## Introduction

The horse is one of the most widely distributed livestock species across the globe owing to their utility in transportation and extensive use in trade and warfare in the past. For landlocked Bhutan characterized by rugged terrain and harsh climatic conditions, the highly adapted traditional horses played an unfailing role in country’s trade. The traditional horses were decsribed as intelligent, enduring and good performers in challenging environment in reports of British expeditions to Bhutan [1]. Apart from mountainous terrain, they also perfromed well in plains as indicated by the demand and export to Assam (a state of India bordering Bhutan). The export of horse topped export earnings from trade with Assam, India during 1907–1926 [2]. Overall, the traditional horses were an important commodity and critical component of trade during the premodernization era.

The estimated horse population of 13,900 [3] in the country is predominantly Yuta (97%) and introduced horses (Boeta, Sharta and Haflinger crosses) comprising less than 3%. The Boeta, Sharta, and Yuta horse are considered traditional breeds for their long history of use in the country. The Yuta have been in use in Bhutan since time immemorial and are believed to be the stabilized cross of horse types from Tibet and India [4,5]. On the other hand, the Boeta and Sharta are horses informally introduced from neighbouring regions of Tibet (China) and Arunachal Pradesh (India) during the trade in recent past. These breeds remain largely localized in Bhutanese districts bordering these countries to this day. Their estimated population sizes in Bhutan are less than 100 head each [6].

The overall horse population in the country has declined at an alarming rate (over 40% in a span of five years) and the risk of reduction to a critical number is immenint [6]. The current situation warrants characterization and strategic management of the horse genetic resources in the country. The genetic characterization entails assessment of genetic diversity, assurance of breed integrity and assignment of individuals to predefined population [7]. Genetic diversity as variation in allelic states and quantitative characters in a population is critical for the survival and production [8]. The genetic variation in livestock species is commonly determined by genotyping of DNA microsatellites.

The genetic diversity of traditional Bhutanese horses has not been assessed using standard microsatellite DNA markers till date. Therefore, our study evaluated their genetic diversity and population structure using a set of 29 DNA microsatellite markers. The findings from the current study fills in important information gap and contribute in the strengthening of conservation and effective management of traditional horse genetic resources in the country.

## Materials and methods

### Ethics statement

The study proposal including the ethical clearance was granted by the Research Committee, National Biodiversity Centre, Serbithang. The procedure of DNA sampling by pulling of hairs was considered non-invasive and a technique requiring minimal animal handling. The approval was granted vide NBC/BRD/7/2015-2016/2469 dated January 15th, 2016.

### Sample collection and DNA extraction

**Fig 1:**
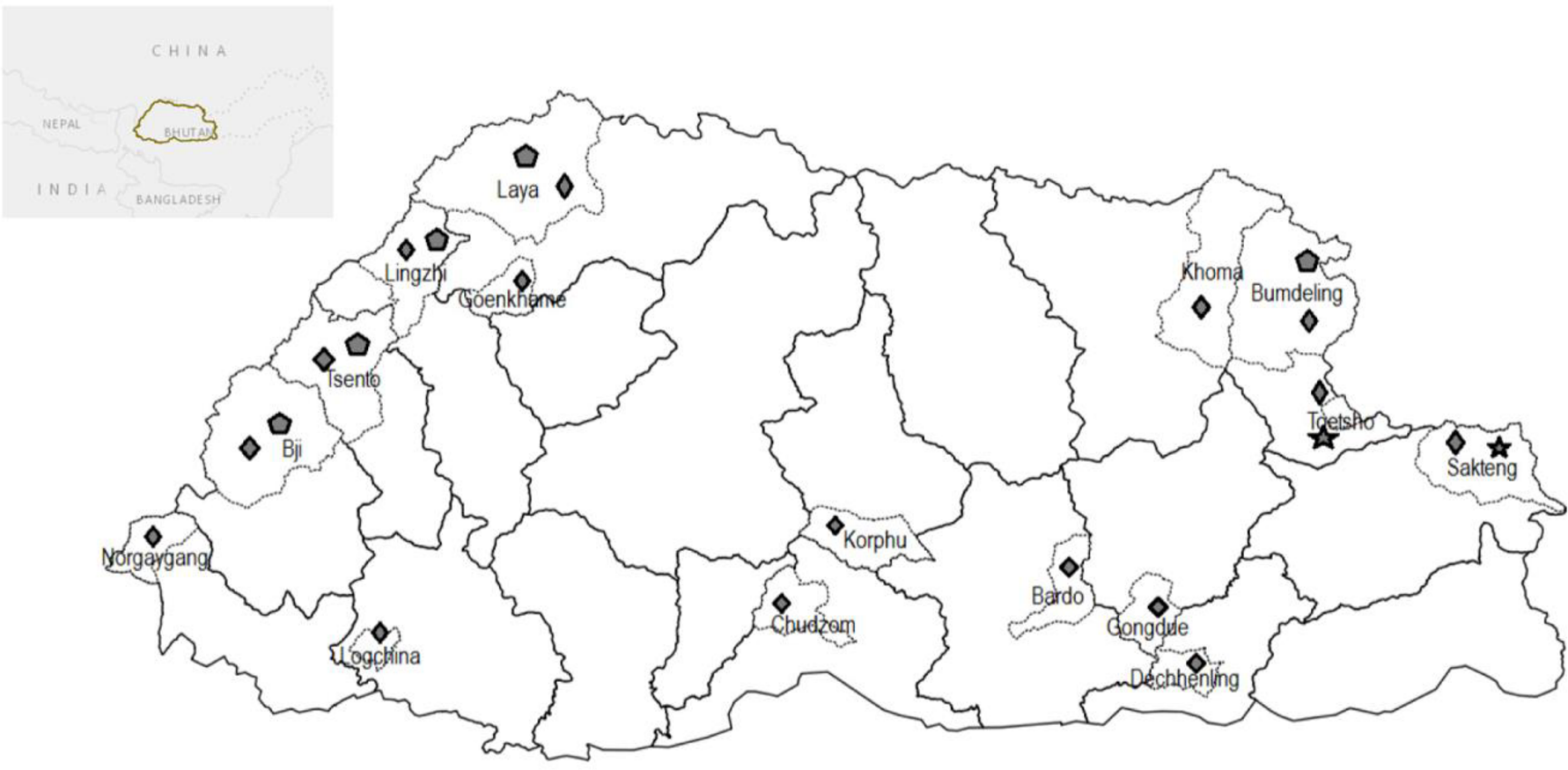
Distribution of traditional horse breeds and sampling sites in Bhutan. ⋆ Sharta, ♦ Yuta and ⬟ Boeta.

Hair samples were plucked from the mane region of horse representative of three traditional breeds (Boeta, Sharta, and Yuta). A purposive sampling was undertaken to cover around 15–20% of the breed populations (Boeta, N = 26; Sharta, N = 15), and 35 Yuta horses from 16 major Yuta horse rearing Gewog (sub-districts) of Bhutan (Fig 1). In all the cases, care was taken to collect a sample from horses which had no parental or sibling relationship based on the mating history recollected by the horse owners. The hair samples were stored in envelopes at room temperature prior to use. The genomic DNA was extracted from the hair follicle following the procedures in Kakoi et al. [9] with slight modifications. Briefly, our procedure used six hairs with roots per sample for incubation in 200 μl extraction buffer (2.5 mmol/L MgCl_2_, 2.5 μg proteinase K2, PCR reaction buffer 3) at 60˚C for 1 hour and then heated to 97˚C in a thermal cycler for 1 hour. The extracts were then stored at 4°C and the stored solution was used directly for the PCR procedure.

### Microsatellite loci and genotyping

We used a set of 29 highly polymorphic microsatellite loci (S1 Table) which were internationally standardized by the International Society for Animal Genetics (ISAG) [10] for evaluation of genetic diversity within and among horse breeds. A multiplex PCR amplification for these DNA markers was carried out according to procedures of Kakoi et al. [11] and Tozaki et al. [12] with minor modification (S2 Table). Genotyping for each marker was performed by using Applied Biosystems 3130xl DNA analyzer (Thermo Fisher Scientific) and GeneMapper® Software 5 (Thermo Fisher Scientific). The accuracy of genotypes was validated by referring to the results of the Horse Comparison Test conducted by ISAG.

### Statistical analysis

The relatedness among the genotyped animals was examined using Coancestry software version 1.0.1.8 [13] to rule out parental and sibling. One of the two closely related Boeta individuals was dropped and final dataset for the analysis consisted of 25 Boeta, 14 Sharta, and 35 Yuta horses. For each locus and breed, the GenAlEx software version 6.5 [14, 15] was used to estimate allele frequencies; the number of observed (*N*_A_) and number of effective alleles (*N*_E_), observed (*H*_O_) and expected (*H*_E_) heterozygosity, and analysis of molecular variance (AMOVA). Polymorphism information content (PIC) of each locus was quantified using Cervus software version 3.0.7 [16]. Further, the genetic differentiation between and within populations was estimated based on unbiased F-statistics according to the method of Weir and Cockerham [17] in FSTAT software version 2.9.3.2 [18]. The exact probability test was performed to determine departure from Hardy-Weinberg equilibrium (HWE) using GENEPOP software version 3.4 [19, 20] from Markov chain algorithm based on 1000 dememorization steps, 100 batches, and 1000 iterations per batch. Sequential Bonferroni correction [21] was applied for all multiple tests performed simultaneously.

The Nei’s genetic distance (*D*_A_) between a pair of breeds was estimated and used to produce a neighbor-joining tree in POPTREEW [22] to visualize genetic relationship. To test if the population has experienced a genetic bottleneck in the recent past, BOTTLENECK version 1.2.02 [23] was used under the assumption of TPM (Two-Phase Mutation) model with SMM set to 0.000 and variance of geometric distribution = 0.036 as recommended for DNA microsatellite. The deviations from the equilibrium were tested using Wilcoxon signed-rank test with *P*<0.05 as the significance level. The genetic bottleneck test was reconfirmed through a Mode shift indicator test based on qualitative descriptive allele frequency distribution.

The structure and extent of admixture of the population were analyzed with STRUCTURE version 2.3.4 [24] using a Bayesian approach. We used an admixture model in which individual may have mixed ancestry. The highest level of population structure; each run (starting with K = 2 to K = 5) was identified using Evanno’s delta K method [25]. We used the burn-in and simulation length of 10000 and 100000 runs respectively to estimate the number of genetic clusters (K). The estimated probabilities and values of log n likelihoods were converted to plots in POPHELPER [26]. The gene flow among the breeds was indirectly calculated from *F*_ST_ in GenAlEx.

## Results

### Genetic diversity

The observed (*N*_A_) and effective number (*N*_E_) of alleles, observed (*H*_O_) and expected (*H*_E_) heterozygosities of the breeds, and polymorphism information content (PIC) values are presented in Table 1. All 29 loci had PIC values above the threshold (>0.5) required for their suitability for genetic studies and ranged from 0.57 (HTG4) to 0.86 (TK333, TK374). A total of 282 alleles were detected across 29 polymorphic loci and three breeds. The *N*_A_ ranged from 5.67 (TKY294) to 12 (ASB17) with an average of 8.06 across the population. By breed, the highest mean *N*_A_ and *N*_E_ estimates were in Yuta followed by Boeta and the least in Sharta. Across the breeds and loci, the *N*_E_ ranged from 1.85 (HTG4, Boeta) to 8.94 (TKY343, Yuta). The mean heterozygosity estimates (*H*_E_ and *H*_O_) were similar in all the breeds.

**Table 1:** The genetic diversity indices for the three traditional horse breeds of Bhutan.

	Overall population					Boeta				Sharta				Yuta			
Locus	*N*_A_	*N*_E_	*H*_O_	*H*_E_	PIC	*N*_A_	*N*_E_	*H*_O_	*H*_E_	*N*_A_	*N*_E_	*H*_O_	*H*_E_	*N*_A_	*N*_E_	*H*_O_	*H*_E_
**AHT4**	8.33	5.60	0.89	0.82	0.81	9.00	5.56	0.92	0.82	7.00	5.30	0.93	0.81	9.00	5.95	0.83	0.83
**AHT5**	6.33	4.90	0.87	0.80	0.78	7.00	5.19	0.76	0.81	5.00	4.72	0.93	0.79	7.00	4.79	0.91	0.79
**ASB17**	12.00	6.18	0.84	0.82	0.84	13.00	6.61	0.96	0.85	9.00	3.96	0.71	0.75	14.00	7.98	0.86	0.87
**ASB2**	8.33	4.87	0.71	0.79	0.78	8.00	5.25	0.76	0.81	7.00	4.96	0.57	0.80	10.00	4.38	0.80	0.77
**ASB23**	8.33	5.45	0.77	0.81	0.78	9.00	4.65	0.60	0.78	6.00	5.37	0.86	0.81	10.00	6.33	0.86	0.84
**CA425**	7.33	4.15	0.82	0.76	0.73	7.00	4.28	0.88	0.77	7.00	4.04	0.86	0.75	8.00	4.13	0.71	0.76
**HMS2**	8.00	4.87	0.82	0.79	0.78	9.00	5.02	0.88	0.80	8.00	4.51	0.86	0.78	7.00	5.07	0.71	0.80
**HMS3**	7.00	3.69	0.81	0.73	0.70	8.00	4.28	0.84	0.77	6.00	3.35	0.79	0.70	7.00	3.43	0.80	0.71
**HMS6**	6.00	4.76	0.84	0.79	0.78	6.00	4.68	0.92	0.79	6.00	4.96	0.93	0.80	6.00	4.63	0.69	0.78
**HMS7**	6.67	3.86	0.69	0.74	0.70	6.00	3.14	0.56	0.68	6.00	4.40	0.79	0.77	8.00	4.04	0.71	0.75
**HTG10**	8.67	3.91	0.74	0.71	0.75	9.00	4.79	0.72	0.79	6.00	2.24	0.64	0.55	11.00	4.69	0.86	0.79
**HTG4**	6.00	2.75	0.57	0.60	0.57	6.00	1.85	0.32	0.46	6.00	3.92	0.79	0.74	6.00	2.49	0.60	0.60
**LEX33**	8.33	5.85	0.83	0.83	0.82	8.00	6.31	0.76	0.84	9.00	5.94	0.93	0.83	8.00	.5.29	0.80	0.81
**TKY19**	7.67	5.35	0.83	0.81	0.80	7.00	4.63	0.80	0.78	7.00	5.37	0.93	0.81	9.00	6.05	0.77	0.83
**TKY279**	6.67	4.13	0.81	0.75	0.75	6.00	3.56	0.76	0.72	6.00	3.50	0.71	0.71	8.00	5.33	0.94	0.81
**TKY287**	9.33	5.99	0.77	0.82	0.80	11.00	5.23	0.80	0.81	9.00	8.17	0.86	0.88	8.00	4.59	0.66	0.78
**TKY294**	5.67	3.72	0.75	0.73	0.70	6.00	3.21	0.80	0.69	5.00	3.96	0.79	0.75	6.00	3.98	0.66	0.75
**TKY301**	8.00	5.29	0.89	0.81	0.80	8.00	5.58	0.96	0.82	8.00	5.52	0.93	0.82	8.00	4.76	0.77	0.79
**TKY312**	9.67	3.96	0.69	0.74	0.72	11.00	3.69	0.68	0.73	9.00	4.72	0.71	0.79	9.00	3.47	0.69	0.71
**TKY321**	9.00	5.30	0.77	0.81	0.80	8.00	5.14	0.68	0.81	8.00	5.03	0.86	0.80	11.00	5.72	0.77	0.83
**TKY325**	9.00	5.85	0.79	0.82	0.85	10.00	6.54	0.76	0.85	7.00	4.26	0.71	0.77	10.00	6.73	0.89	0.85
**TKY333**	9.33	6.33	0.82	0.83	0.86	11.00	6.94	0.88	0.86	6.00	4.26	0.79	0.77	11.00	7.78	0.80	0.87
**TKY337**	7.33	6.03	0.83	0.83	0.83	8.00	6.38	0.92	0.84	6.00	5.76	0.71	0.83	8.00	5.95	0.86	0.83
**TKY341**	6.67	4.61	0.72	0.78	0.78	7.00	4.56	0.80	0.78	6.00	4.22	0.64	0.76	7.00	5.04	0.71	0.80
**TKY343**	10.33	6.17	0.82	0.82	0.85	14.00	5.84	0.72	0.83	6.00	3.73	0.86	0.73	11.00	8.94	0.89	0.89
**TKY344**	7.67	3.31	0.74	0.69	0.67	8.00	3.29	0.72	0.70	7.00	3.73	0.79	0.73	8.00	2.91	0.71	0.66
**TKY374**	9.67	7.23	0.87	0.86	0.86	10.00	6.76	0.88	0.85	8.00	7.40	0.86	0.86	11.00	7.54	0.89	0.87
**TKY394**	7.67	3.54	0.61	0.69	0.72	8.00	3.50	0.56	0.71	6.00	2.24	0.43	0.55	9.00	4.89	0.83	0.80
**VHL20**	8.67	6.33	0.89	0.84	0.84	9.00	6.65	0.84	0.85	7.00	5.44	0.93	0.82	10.00	6.90	0.91	0.86
**Mean**	**8.06**	**4.96**	**0.79**	**0.78**	**0.77**	**8.52**	**4.94**	**0.77**	**0.78**	**6.86**	**4.65**	**0.80**	**0.77**	**8.79**	**5.30**	**0.79**	**0.79**
**SE**	**0.27**	**0.21**	**0.01**	**0.01**	**0.01**	**0.38**	**0.24**	**0.03**	**0.01**	**0.22**	**0.23**	**0.02**	**0.01**	**0.35**	**0.29**	**0.02**	**0.01**

*N*_A_ Number of alleles observed, *N*_E_ number of effective alleles, *H*_O_ observed heterozygosity,

*H*_E_ expected heterozygosity, PIC polymorphism information content

The coefficient of inbreeding (*F*_IS_) estimate was low (0.027, 0.001 and 0.021 for Boeta, Sharta, and Yuta respectively) and not significantly different from zero (p <0.05). There was no significant deviation from Hardy-Weinberg equilibrium following sequential Bonferroni correction. The total genetic variation was mainly within individual variation (97.5%) (p<0.05), and 1.9% (p = 0.056) and 0.6% (p<0.05) among individuals and among the breeds respectively from the analysis of the molecular variance.

**Fig 2:**
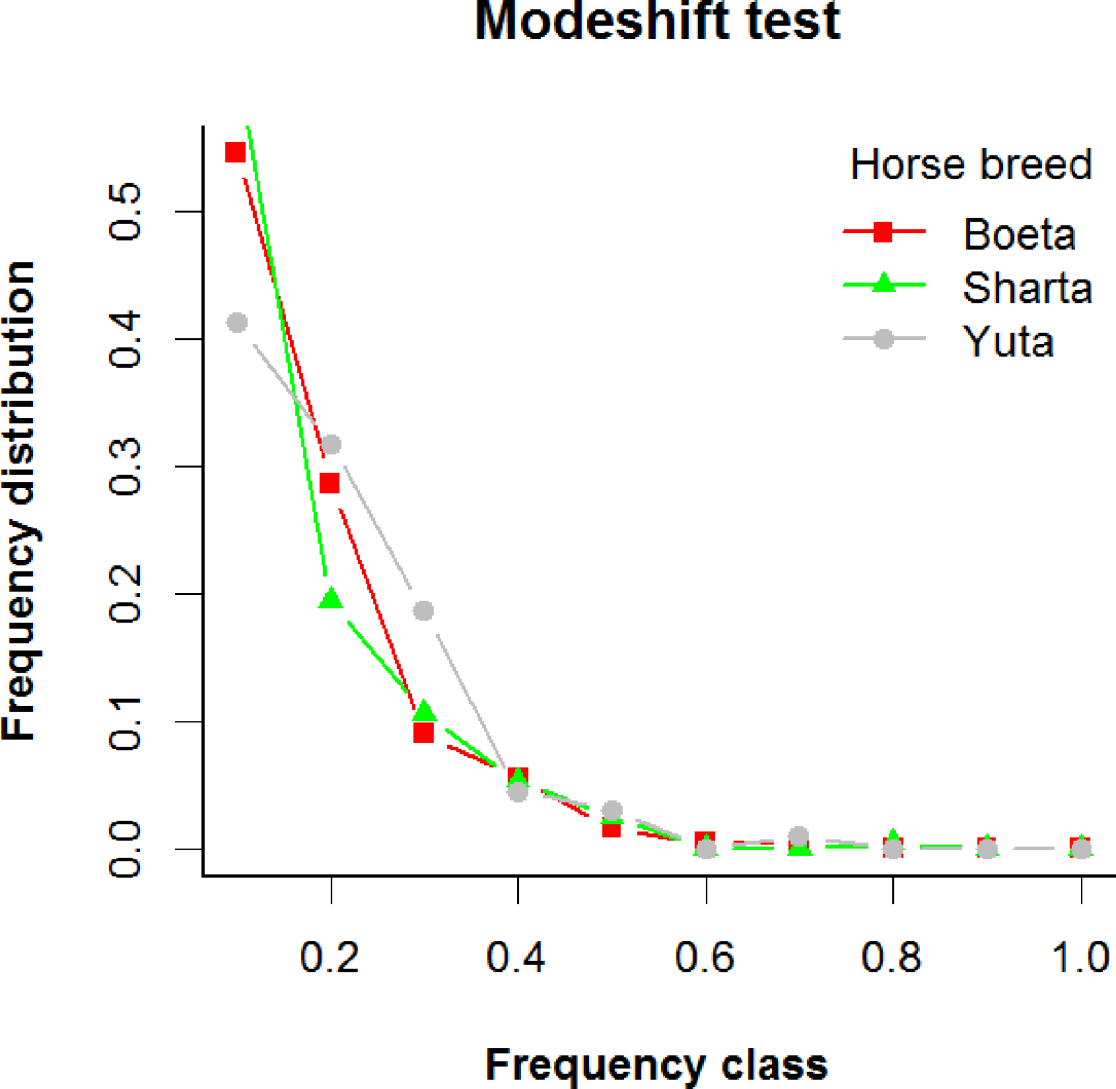
Mode-shift analysis of genetic bottleneck in Bhutanese traditional horse breeds.

The test for genetic bottleneck did not detect significant bottleneck effects at p<0.05 for all breed populations (p = 0.877 Boeta, 0.282 Sharta, 0.551 Yuta). According to the test, a population which experienced a bottleneck exhibits a significant heterozygosity excess. This result was supported by mode-shift indicator test in which all the breed population at equilibrium shows a normal “L” shaped allele frequency distribution (Fig 2). Altogether, these results suggest the traditional horse breeds may not have experienced serious demographic bottlenecks in the recent past.

### Breed relationship

**Fig 3:**
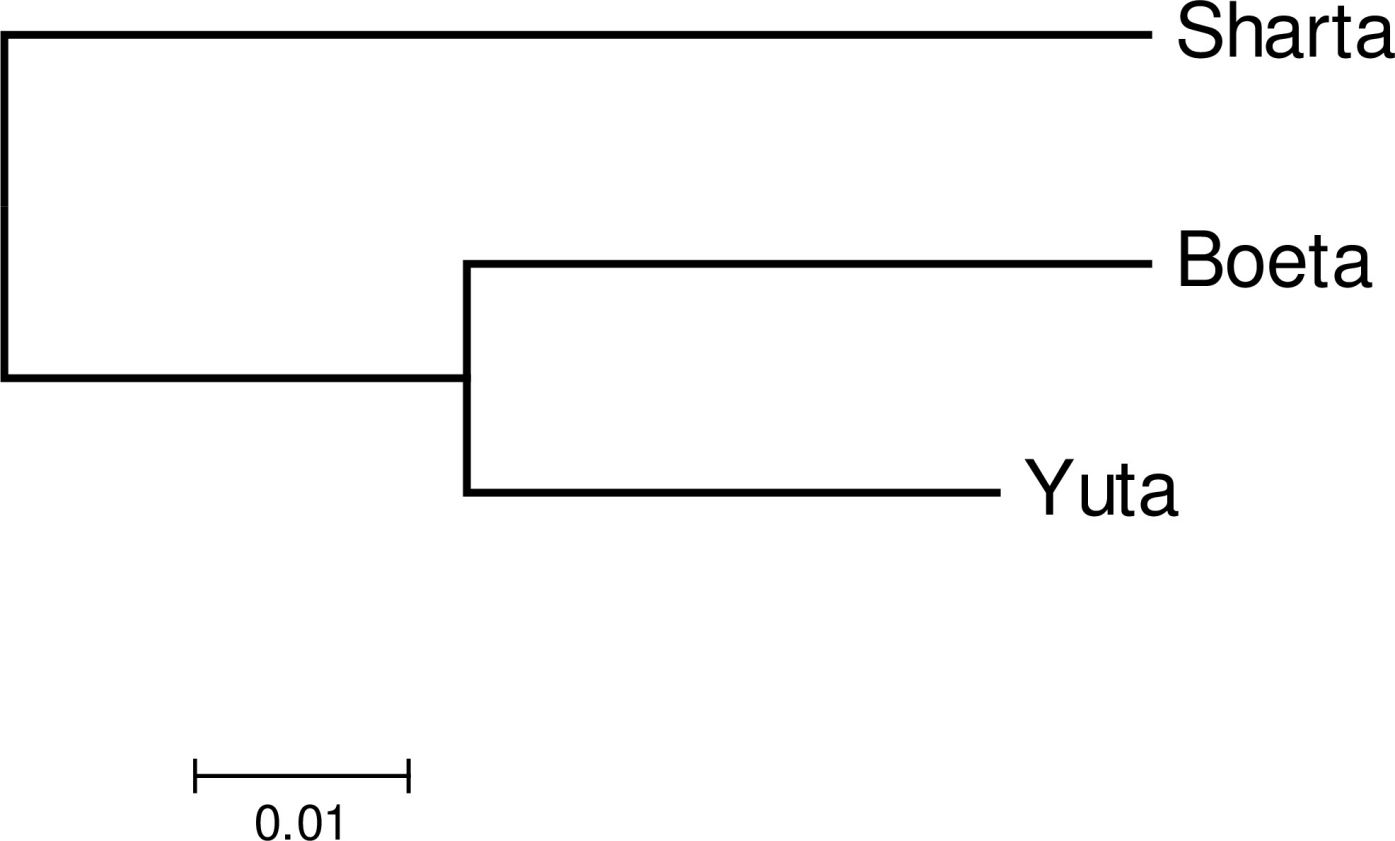
The neighbor-joining tree showing the level of genetic relationships among three traditional horse breeds of Bhutan based on Nei’s genetic distances (*D*_A_).

The genetic differentiation (*F*_ST_) among the breeds was low. The F-statistics obtained by jackknifing over loci was *F*_IT_ (0.025), *F*_ST_ (0.006) and *F*_IS_ (0.019). The genetic differentiation between breeds based on Nei’s unbiased genetic distance (*D*_A_) and population differentiation (*F*_ST_) between breed pairs (Table 2) suggest Boeta and Yuta are genetically less differentiated breeds compared to other breed combinations (Fig 3)

**Table 2:** Nei’s unbiased genetic distance (*D*_A_) below diagonal and pairwise population differentiation (*F*_ST_) above the diagonal among three breeds.

Breeds	Boeta	Sharta	Yuta
**Boeta**	-	0.008	0.003
**Sharta**	0.114	-	0.008
**Yuta**	0.079	0.117	-

**Fig 4:**
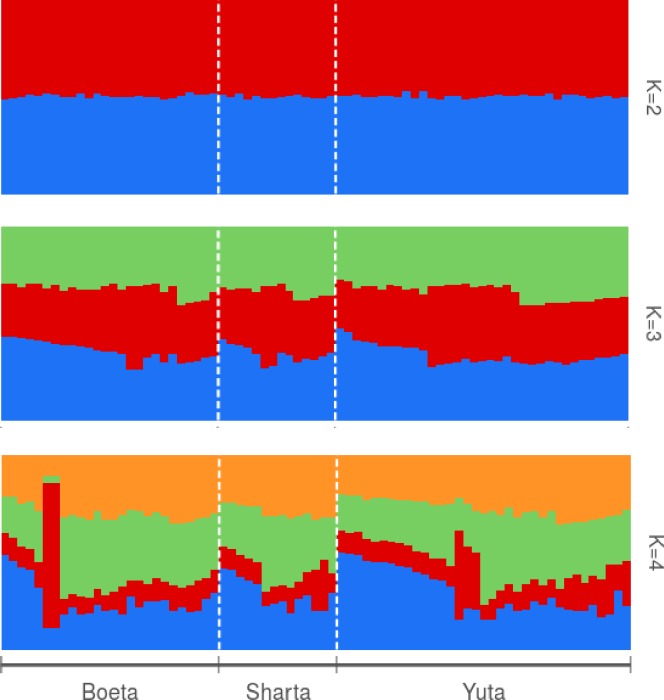
Population structure of traditional horses population of Bhutan for *K* = 2 to *K* = 4.

The examination of population structure of Bhutanese traditional horse breeds through STRUCTURE program suggested a lack of definite structure (Fig 4) although Evanno’s test indicated that the most informative number of subpopulations was *K* = 3 (S1 Fig). This suggested a high genetic exchange among the breeds and is also supported by gene flow analysis. The estimated amount of gene flow among the population indirectly calculated from *F*_*ST*_ was high (45.6) with highest between Boeta and Yuta (73.9) followed by Sharta-Yuta (32.3) and the least (30.6) in Boeta-Sharta pairs.

## Discussion

The high PIC values of all 29 microsatellite loci used in this study showed that the markers were suitable for genetic evaluation of Bhutanese traditional horse breeds. Overall, the high genetic diversity of the traditional horse population as assessed through standard diversity indices are optimistic for the resilience to environmental changes in future. In general, the adjusted genetic diversity of Bhutanese traditional horses for the common loci was high and comparable to American horses’ study involving 50 breeds [27] (mean number of alleles MNA 7.5; *H*_O_ 0.74, *H*_E_ 0.75, *F*_IS_ 0.02) (S3 Table) and five Indian native horses (MNA 14; *N*_E_ 6.62; *H*_O_ 0.72) [28] for 24 common loci (S4 Table). By breed, the genetic diversity of Bhutanese traditional horses was similar to the native Korean Halla horse [29] (S7 Table) but higher than endangered Japanese breeds (S5 and S6 Tables) as expected; Yonaguni horse (*N*_A_ 4.3; *H*_O_ 0.59; *H*_E_ 0.6) [30], Kiso horse (*N*_A_ 6.3, *H*_O_ 0.66, *H*_E_ 0.65) [31] and Miyako (*N*_A_ 4.2; *H*_O_ 0.70; *H*_E_ 0.649) [32].

There was no evidence of a genetic bottleneck in all traditional breeds. A recent genetic bottleneck is an important aspect to consider for conservation because it results in a loss of genetic variability, inbreeding, and expression of undesirable recessive alleles, and therefore reduce survival rates. As such the population of the horse in the country has not declined drastically until the last decade [33]. It is interesting to note the absence of signs of a genetic bottleneck in Boeta and Sharta despite the small populations in the country. This result in part may be explained by potential interbreeding among the breeds owing to the lack of isolation.

The Yuta horses were considered as inbred in the recent past (based on phenotypic observations) [4,5]. The current study employing molecular technique confirms the inbreeding in Yuta populations but finds it low and within the acceptable levels. One of the key factors attributing to the low level of inbreeding may be due to random mating. The random mating is favoured by the prevailing horse breeding environment in the country characterized by lack of selective breeding by farmers and want of Yuta horse breed improvement program in the country.

The levels of genetic differentiation (*F*_ST_) in this study corresponds to the previously reported estimates both within subpopulations of a breed as wells as between breeds. The *F*_ST_ estimates for the subpopulations of the Nordestino horse was 0.005 [34] and Thoroughbred populations of UK and US was 0.004 [35]. Among the breeds, *F*_ST_ as low as 0.001 (Guizou and Luoping horses) [36], 0.002 (Paint and Quarter horses), and 0.006 in Tuva and Mongolian horses [35] are reported.

The lack of population structure and high gene flow among the traditional breeds in this study also suggests the potential interbreeding among the breeds. Further, the absence of salient distinguishing phenotypes among the traditional breeds [4,6] supports the probability of being a subpopulation of a breed. The breed is named based on morphological differences and/or in relation to geographical locations and thereby different names are assigned to very closely related populations located in different administrative areas [37]. Similarly, the current study also utilized the breed classification based on source country of the horses.

In summary, this study suggests the traditional horse breeds of Bhutan are genetically less differentiated. Nonetheless, a high individual genetic diversity provides an optimistic outlook for the survival of the declining traditional horse population with appropriate management interventions.

## Supporting information

S1 Fig Delta K revealing the true values of K. (TIF)

S1 Table Information on the set of marker used in the study. (XLSX)

S2 Table Multiplex PCR groups. (XLS)

S3 Table Bhutanese traditional horses adjusted to common loci with American breeds. (XLSX)

S4 Table Bhutanese and five Indian horse breeds adjusted to common loci. (XLSX)

S5 Table Yunagoni horse, Japan. (XLSX)

S6 Table Kiso horse, Japan. (XLSX)

S7 Table Halla horse, Korea. (XLSX)

S1 Text DNA extraction from hair roots. (TXT)

S2 Text HWE estimation of exact P value. (TXT)

S3 Text Summary of bottleneck analysis. (TXT)

S4 Text Number of migrants based on private alleles method. (TXT)

S5 Text Overall population F statistics. (TXT)
