# Supplementary figures and images for: Genetic diversity and population structure of three traditional horse breeds of Bhutan based on 29 DNA microsatellite markers
Source: PLoS One. 2018 Jun 27;13(6):e0199376. doi: 10.1371/journal.pone.0199376 (PMC6021118; doi:10.1371/journal.pone.0199376)

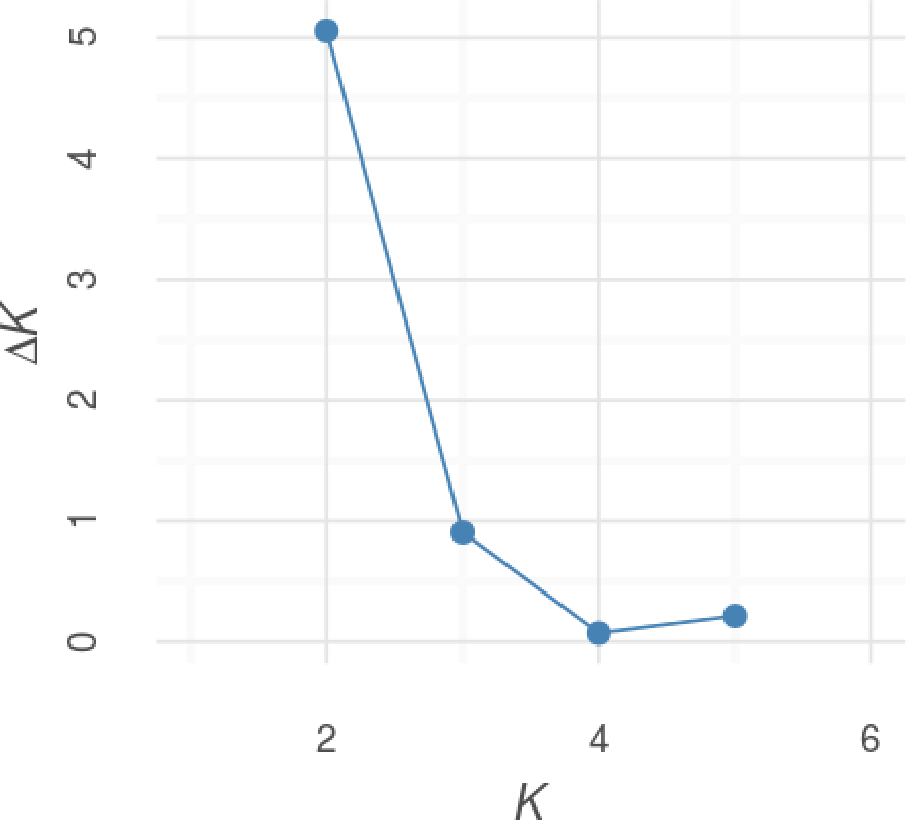

Supplement: S1 Fig — (TIF) [file pone.0199376.s001.tif]
